# Supplementary material for: Direct activation of a bacterial innate immune system by a viral capsid protein
Source: Nature. 2022 Nov 16;612(7938):132–40. doi: 10.1038/s41586-022-05444-z (PMC9712102; doi:10.1038/s41586-022-05444-z)
Supplement: Supplementary file 1 — This file contains Supplementary Fig. 1 and Tables 1–4. Supplementary Fig. 1: uncropped blots from main figures and extended data figures. a, Immunoblot of His6–CapRelSJ46 for Fig. 3a. b, Coomassie stain shown in Extended Data Fig. 4b as a loading control for Fig. 3a. c, Blue silver stain of in vitro transcription–translation assays for Fig. 3j. d, Autoradiography and SYBR Gold stain for Fig. 3k. e, Immunoblots of lysates and IP samples for Fig. 4a. f, Immunoblots of lysates and IP samples for Fig. 4h. g, Immunoblot for Extended Data Fig. 3c. h, Immunoblot of His6–CapRelSJ46 for Extended Data Fig. 4c and the corresponding loading control. i, Immunoblots of Gp57–HA for Extended Data Fig. 4l and the corresponding loading controls. 3 independent replicates are shown. Supplementary Table 1: list of all mutations present in the phage genomes of 10 evolved clones from 5 independent populations or 2 clones from the control population. Supplementary Table 2: list of bacterial and phage strains used in this study. Supplementary Table 3: list of plasmids used in this study. Supplementary Table 4: list of primers and synthesized gene sequences used in this study. [file 41586_2022_5444_MOESM1_ESM.pdf]

nature portfolio

https://doi.org/10.1038/s41586-022-05444-z

Supplementary information

Direct activation of a bacterial innate immune system by a viral capsid protein

In the format provided by the authors and unedited

Supplementary Figure 1: raw gels

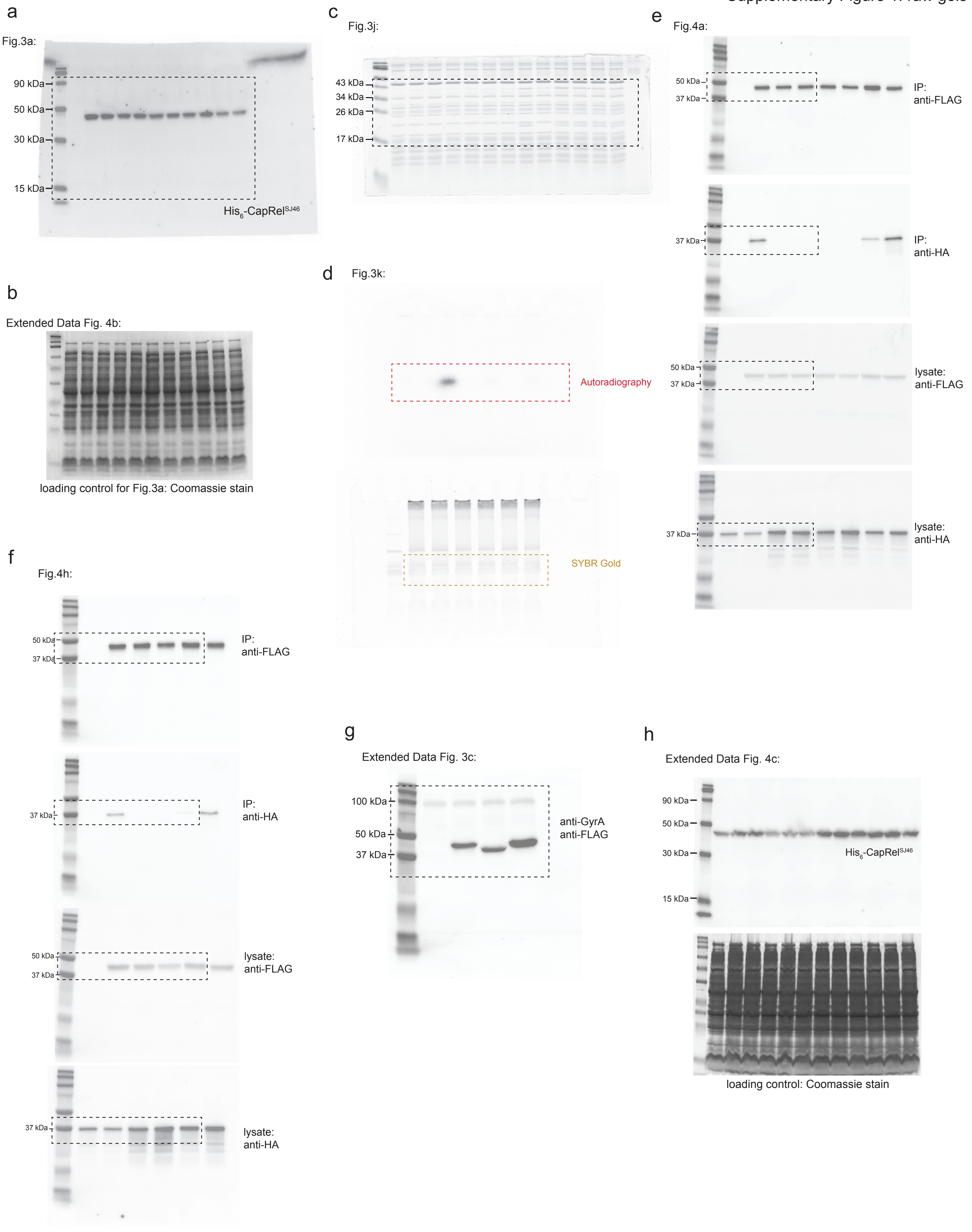

i

Extended Data Fig. 4l:

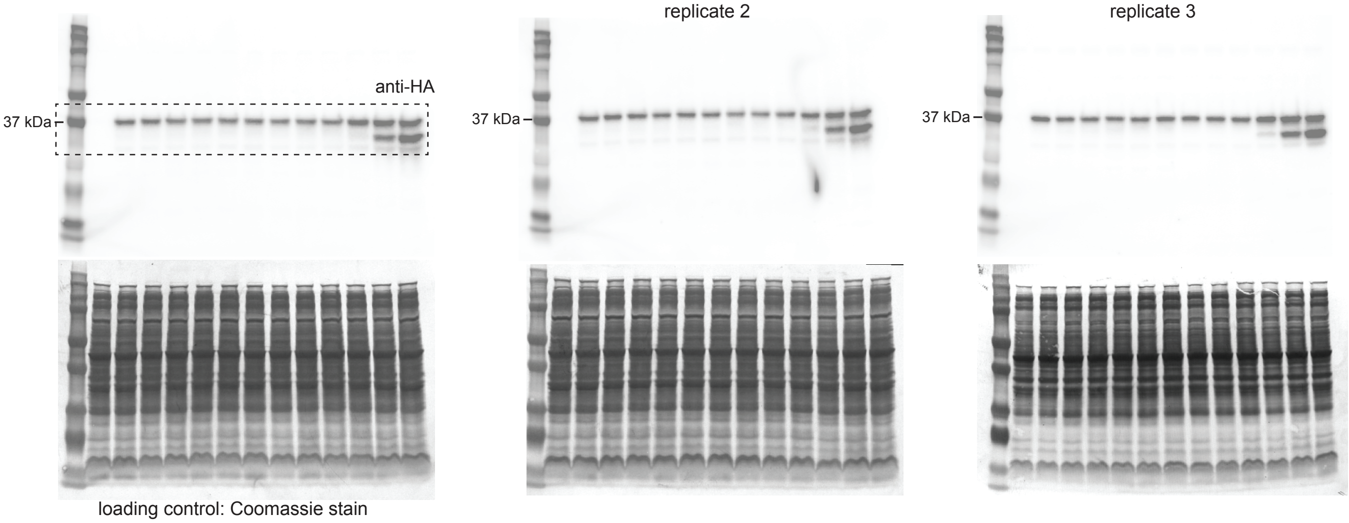

SI Table 1. Mutations present in the phage genomes of 10 evolved clones from 5 independent populations or 2 clones from the control population.

phage clone	mutated DNA	mutated protein	mutations
control clone 1	C 40530 T	Gp72 hypothetical protein	E163K
control clone 2	G 23733 T<br>C 40530 T	Gp52 phage portal protein<br>Gp72 hypothetical protein	A398S<br>E163K
population 1 clone 1	A 27499 T<br>G 1536 T	Gp57 major capsid protein<br>hypothetical protein	I115F<br>E19D
population 1 clone 2	T 27497 C	Gp57 major capsid protein	L114P
population 2 clone 1	T 27497 C	Gp57 major capsid protein	L114P
population 2 clone 2	T 27497 C<br>A 41518 G<br>T 23574 C	Gp57 major capsid protein<br>Gp74 hypothetical protein<br>Gp52 phage portal protein	L114P<br>C17R<br>F345L
population 3 clone 1	T 27497 C	Gp57 major capsid protein	L114P
population 3 clone 2	T 27497 C<br>G 23533 A	Gp57 major capsid protein<br>Gp52 phage portal protein	L114P<br>R331Q
population 4 clone 1	T 27497 C	Gp57 major capsid protein	L114P
population 4 clone 2	T 27497 C<br>A 44962 C	Gp57 major capsid protein<br>hypothetical protein	L114P<br>M201R
population 5 clone 1	T 27497 C	Gp57 major capsid protein	L114P
population 5 clone 2	T 27497 C	Gp57 major capsid protein	L114P

SI Table 2. Strains

Bacterial Strains

**Name**	**Genotype**	**Source**
ML6	MG1655	
	DH5α	Invitrogen
ML3836	BW27783	
	BL21 (DE3)	Lab stock
ML3837	MG1655 pBAD33-EV pEXT20-EV	this study
ML3838	MG1655 pBAD33-*capRel*^{SJ46}*(1-272)* pEXT20-EV	this study
ML3839	MG1655 pBAD33-*capRel*^{SJ46}*(1-272)* pEXT20-*capRel*^{SJ46}*(273-373)*	this study
ML3840	MG1655 pBR322-EV	this study
ML3841	MG1655 pBR322-*capRel*^{SJ46}	this study
ML3842	MG1655 pBR322-*capRel*^{Ebc}	this study
ML3843	MG1655 pBR322-*capRel*^{Kp}	this study
ML3844	MG1655 pBR322-*capRel*^{SJ46}*(Y155A)*	this study
ML3845	MG1655 pBR322-*capRel*^{Ebc}*(Y153A)*	this study
ML3846	MG1655 pBR322-*capRel-chimera*	this study
ML3847	MG1655 pBAD33-*capRel*^{SJ46}	this study
ML3848	MG1655 pBAD33-*capRel*^{SJ46}*(A77K)*	this study
ML3849	MG1655 pBAD33-*capRel*^{SJ46}*(R116A)*	this study
ML3850	MG1655 pBAD33-*capRel*^{SJ46}*(V338A)*	this study
ML3851	MG1655 pBAD33-*capRel*^{SJ46}*(L339A)*	this study
ML3852	MG1655 pBAD33-*capRel*^{SJ46}*(A341K)*	this study
ML3853	MG1655 pBAD33-*capRel*^{SJ46}*(A351K)*	this study
ML3854	MG1655 pBAD33-*capRel*^{SJ46}*(Y352A)*	this study
ML3855	MG1655 pBAD33-*capRel*^{SJ46}*(Y355A)*	this study
ML3856	MG1655 pBR322-*His*$_6$*-capRel*^{SJ46}	this study
ML3857	MG1655 pBR322-*capRel*^{SJ46} pBAD33-EV	this study
ML3858	MG1655 pBR322-*capRel*^{SJ46} pBAD33-*gp57*	this study
ML3859	MG1655 pBR322-*capRel*^{SJ46} pBAD33-*gp57(L114P)*	this study
ML3860	MG1655 pBR322-*capRel*^{SJ46} pBAD33-*gp57(I115F)*	this study
ML3861	MG1655 pBR322-*capRel*^{SJ46} pBAD33-*gp57-HA*	this study
ML3862	MG1655 pBR322-*capRel*^{SJ46}*-FLAG* pBAD33-*gp57-HA*	this study
ML3863	MG1655 pBR322-*capRel*^{SJ46}*-FLAG* pBAD33-*gp57(L114P)-HA*	this study
ML3864	MG1655 pBR322-*capRel*^{SJ46}*-FLAG* pBAD33-*gp57(I115F)-HA*	this study
ML3865	MG1655 pBR322-EV pBAD33-*gp57*	this study
ML3866	MG1655 pBR322-*capRel*^{SJ46}*(L280Q)* pBAD33-*gp57*	this study
ML3867	MG1655 pBR322-*capRel*^{SJ46}*(L280P)* pBAD33-*gp57*	this study
ML3868	MG1655 pBR322-*capRel*^{SJ46}*(L307A)* pBAD33-*gp57*	this study
ML3869	MG1655 pBR322-*capRel*^{SJ46}*(L280Q)*	this study
ML3870	MG1655 pBR322-*capRel*^{SJ46}*(L280P)*	this study
ML3871	MG1655 pBR322-*capRel*^{SJ46}*(L307A)*	this study
ML3872	MG1655 pBR322-*capRel*^{SJ46}*(L280Q)-FLAG* pBAD33-*gp57-HA*	this study

ML3873	MG1655 pBR322-*capRel*^{SJ46}*(L280P)-FLAG* pBAD33-*gp57-HA*	this study
ML3874	MG1655 pBR322-*capRel*^{SJ46}*(L307A)-FLAG* pBAD33-*gp57-HA*	this study
ML3875	MG1655 pBR322-*capRel*^{SJ46} pBAD33-*gp8*^{Bas4}	this study
ML3876	MG1655 pBR322-*capRel*^{SJ46} pBAD33-*gp8*^{Bas5}	this study
ML3877	MG1655 pBR322-*capRel*^{SJ46} pBAD33-*gp8*^{Bas8}	this study
ML3878	MG1655 pBR322-*capRel*^{SJ46} pBAD33-*gp8*^{Bas4}*(Y113F)*	this study
ML3879	MG1655 pBR322-*capRel*^{SJ46} pBAD33-*gp57(F113Y)*	this study
ML3880	MG1655 pBR322-*capRel*^{SJ46}*-FLAG*	this study
ML3881	MG1655 pBR322-EV pBAD33-EV	this study
ML3882	MG1655 pBR322-EV pBAD33-*gp57*	this study
ML3883	MG1655 pBR322-EV pBAD33-*gp57(L114P)*	this study
ML3884	MG1655 pBR322-EV pBAD33-*gp57(I115F)*	this study
ML3885	MG1655 pBR322-*capRel*^{SJ46}*(Y155A)* pBAD33-EV	this study
ML3886	MG1655 pBR322-*capRel*^{SJ46}*(Y155A)* pBAD33-*gp57*	this study
ML3887	MG1655 pBR322-*capRel*^{SJ46}*(Y155A)* pBAD33-*gp57(L114P)*	this study
ML3888	MG1655 pBR322-*capRel*^{SJ46}*(Y155A)* pBAD33-*gp57(I115F)*	this study
ML3889	MG1655 pBAD33-EV	this study
ML3890	MG1655 pBAD33-*capRel*^{SJ46}*(1-272)*	this study
ML3891	MG1655 pBR322-EV pBAD33-*gp57-HA*	this study
ML3892	MG1655 pBR322-*capRel*^{SJ46}*-FLAG* pBAD33-*gp57*	this study
ML3893	BW27783 pBR322-EV pBAD33-*gp8*^{Bas8}	this study
ML3894	BW27783 pBR322-*capRel*^{SJ46} pBAD33-*gp8*^{Bas8}	this study
ML3895	BW27783 pBR322-*capRel*^{SJ46} pBAD33-*gp8*^{Bas8}*(F120L)*	this study
ML3896	BW27783 pBR322-*capRel*^{SJ46} pBAD33-*gp8*^{Bas8}*(I124F)*	this study
ML3897	DH5α pBAD33-EV	this study
ML3898	DH5α pBAD33-*capRel*^{SJ46}*(1-272)*	this study
ML3899	DH5α pEXT20-EV	this study
ML3900	DH5α pEXT20-*capRel*^{SJ46}*(273-373)*	this study
ML3901	DH5α pBR322-EV	this study
ML3902	DH5α pBR322-*capRel*^{SJ46}	this study
ML3903	DH5α pBR322-*capRel*^{SJ46}*(Y155A)*	this study
ML3904	DH5α pBR322-*capRel*^{Ebc}	this study
ML3905	DH5α pBR322-*capRel*^{Ebc}*(Y153A)*	this study
ML3906	DH5α pBR322-*capRel*^{Kp}	this study
ML3907	DH5α pBR322-*capRel-chimera*	this study
ML3908	DH5α pBAD33-*capRel*^{SJ46}	this study
ML3909	DH5α pBAD33-*capRel*^{SJ46}*(A77K)*	this study
ML3910	DH5α pBAD33-*capRel*^{SJ46}*(R116A)*	this study
ML3911	DH5α pBAD33-*capRel*^{SJ46}*(V338A)*	this study
ML3912	DH5α pBAD33-*capRel*^{SJ46}*(L339A)*	this study
ML3913	DH5α pBAD33-*capRel*^{SJ46}*(A341K)*	this study
ML3914	DH5α pBAD33-*capRel*^{SJ46}*(A351K)*	this study
ML3915	DH5α pBAD33-*capRel*^{SJ46}*(Y352A)*	this study
ML3916	DH5α pBAD33-*capRel*^{SJ46}*(Y355A)*	this study
ML3917	DH5α pBR322-*His*$_6$*-capRel*^{SJ46}	this study
ML3918	DH5α pBAD33-*gp57*	this study

ML3919	DH5α pBAD33-*gp57(L114P)*	this study
ML3920	DH5α pBAD33-*gp57(I115F)*	this study
ML3921	DH5α pBR322-*capRel*^{SJ46}*-FLAG*	this study
ML3922	DH5α pBAD33-*gp57-HA*	this study
ML3923	DH5α pBAD33-*gp57(L114P)-HA*	this study
ML3924	DH5α pBAD33-*gp57(I115F)-HA*	this study
ML3925	DH5α pBR322-*capRel*^{SJ46}*(L280Q)*	this study
ML3926	DH5α pBR322-*capRel*^{SJ46}*(L280P)*	this study
ML3927	DH5α pBR322-*capRel*^{SJ46}*(L307A)*	this study
ML3928	DH5α pBR322-*capRel*^{SJ46}*(L280Q)-FLAG*	this study
ML3929	DH5α pBR322-*capRel*^{SJ46}*(L280P)-FLAG*	this study
ML3930	DH5α pBR322-*capRel*^{SJ46}*(L307A)-FLAG*	this study
ML3931	DH5α pBAD33-*gp8*^{Bas4}	this study
ML3932	DH5α pBAD33-*gp8*^{Bas5}	this study
ML3933	DH5α pBAD33-*gp8*^{Bas8}	this study
ML3934	DH5α pBAD33-*gp8*^{Bas4}*(Y113F)*	this study
ML3935	DH5α pBAD33-*gp57(F113Y)*	this study
ML3936	DH5α pBAD33-*gp8*^{Bas8}*(F120L)*	this study
ML3937	DH5α pBAD33-*gp8*^{Bas8}*(I124F)*	this study
ML3938	MG1655 pBAD33-*capRel*^{SJ46}*(R78A)*	this study
ML3939	MG1655 pBAD33-*capRel*^{SJ46}*(K311A)*	this study
ML3940	MG1655 pBAD33-*capRel*^{SJ46}*(R314A)*	this study
ML3941	MG1655 pBAD33-*capRel*^{SJ46}*(E319A)*	this study
ML3942	MG1655 pBAD33-*capRel*^{SJ46}*(K346A)*	this study
ML3943	DH5α pBAD33-*capRel*^{SJ46}*(R78A)*	this study
ML3944	DH5α pBAD33-*capRel*^{SJ46}*(K311A)*	this study
ML3945	DH5α pBAD33-*capRel*^{SJ46}*(R314A)*	this study
ML3946	DH5α pBAD33-*capRel*^{SJ46}*(E319A)*	this study
ML3947	DH5α pBAD33-*capRel*^{SJ46}*(K346A)*	this study
AGP-1	BL21 (DE3) pET21a-*His*$_{10}$*-SUMO-gp57*	this study
AGP-2	BL21 (DE3) pET24d-*N-His*$_{10}$*-SUMO-capRel*^{SJ46}	this study
ML3948	MG1655 chromosomal *capRel*^{SJ46}	this study
ML3949	MG1655 chromosomal *capRel*^{SJ46}*(Y155A)*	this study
ML3950	MG1655 chromosomal *His*$_{6}$*-capRel*^{SJ46}	this study
ML3951	MG1655 pBR322-*capRel*^{Ebc}*-FLAG*	this study
ML3952	MG1655 pBR322-*chimera-FLAG*	this study
ML3953	MG1655 chromosomal *capRel*^{SJ46} pBAD33-EV	this study
ML3954	MG1655 chromosomal *capRel*^{SJ46} pBAD33-*gp57*	this study
ML3955	MG1655 chromosomal *gp57-HA*	this study
ML3956	BW27783 pBR322-EV pBAD33-*gp8*^{Bas8}	this study
ML3957	BW27783 pBR322- EV pBAD33-*gp8*^{Bas8}*(F120L)*	this study
ML3958	BW27783 pBR322- EV pBAD33-*gp8*^{Bas8}*(I124F)*	this study
ML3959	MG1655 pBR322-*capRel*^{EcHT}	this study
ML3960	DH5α pBR322-*capRel*^{Ebc}*-FLAG*	this study
ML3961	DH5α pBR322-*chimera-FLAG*	this study
ML3962	DH5α pBR322-*capRel*^{EcHT}	this study

Phage Strains

Name	Genotype	Source
phML1	T2	ATCC Cat #: 11303-B2
phML2	T3	ATCC Cat #: 11303-B3
phML3	T4	ATCC Cat #: 11303-B4
phML4	T5	ATCC Cat #: 11303-B5
phML5	T6	ATCC Cat #: 11303-B6
phML6	T7	Gift from R. Sorek
phML7	RB69	Laval Collection, HER #158
phML8	λ_{vir}	Gift from R. Sorek
phML9	SECΦ17	(Doron et al., 2018)
phML10	SECΦ18	(Doron et al., 2018)
phML11	SECΦ27	(Doron et al., 2018)
phML12	Lust	(Malki et al., 2016)
phML43	SECΦ27 evolved clone 1 from population 3 containing Gp57(L114P)	this study
phML44	SECΦ27 evolved clone 1 from population 1 containing Gp57(I115F)	this study
phML45	Bas4	(Maffei et al., 2021)
phML46	Bas5	(Maffei et al., 2021)
phML47	Bas8	(Maffei et al., 2021)
phML48	Bas8 escape clone 1 containing Gp8(F120L)	this study
phML49	Bas8 escape clone 2 containing Gp8(I124F)	this study
phML50	Bas4 clone 1 containing Gp8(Y113F)	this study
phML51	Bas4 clone 2 containing Gp8(Y113F)	this study
phML52	Bas8 escape clone containing Gp8(G111S)	this study
phML53	Bas8 escape clone containing Gp8(I124N)	this study
phML54	Bas8 escape clone containing Gp8(L116F)	this study

SI Table 3. Plasmids

**Plasmid**	**Description**	**Source**
pBAD33-EV	empty vector of pBAD33 (p15A ori, P_{ara} promoter)	lab stock
pBAD33-*capRel*^{SJ46}*(1-272)*	arabinose inducible N-terminal fragment (1-272) of CapRel^{SJ46}	this study
pEXT20-EV	empty vector of pEXT20 (pBR322 ori, P_{lac} promoter)	lab stock
pEXT20-*capRel*^{SJ46}*(273-373)*	IPTG inducible C-terminal fragment (273-373) of CapRel^{SJ46}	this study
pBR322-EV	derivative of pBR322 with P_{tet} removed	lab stock
pBR322-*capRel*^{SJ46}	*capRel*^{SJ46} with native promoter	this study
pBR322-*capRel*^{SJ46}*(Y155A)*	*capRel*^{SJ46}*(Y155A)* with native promoter	this study
pBR322-*capRel*^{Ebc}	*capRel*^{Ebc} with native promoter	this study
pBR322-*capRel*^{Ebc}*(Y153A)*	*capRel*^{Ebc}*(Y153A)* with native promoter	this study
pBR322-*capRel*^{Kp}	*capRel*^{Kp} with native promoter	this study
pBR322-*capRel-chimera*	*capRel* chimera producing CapRel^{SJ46} (272-341) replaced with CapRel^{Ebc} (270-339)	this study
pBAD33-*capRel*^{SJ46}	arabinose inducible *capRel*^{SJ46}	this study
pBAD33-*capRel*^{SJ46}*(A77K)*	arabinose inducible *capRel*^{SJ46}*(A77K)*	this study
pBAD33-*capRel*^{SJ46}*(R116A)*	arabinose inducible *capRel*^{SJ46}*(R116A)*	this study
pBAD33-*capRel*^{SJ46}*(V338A)*	arabinose inducible *capRel*^{SJ46}*(V338A)*	this study
pBAD33-*capRel*^{SJ46}*(L339A)*	arabinose inducible *capRel*^{SJ46}*(L339A)*	this study
pBAD33-*capRel*^{SJ46}*(A341K)*	arabinose inducible *capRel*^{SJ46}*(A341K)*	this study
pBAD33-*capRel*^{SJ46}*(A351K)*	arabinose inducible *capRel*^{SJ46}*(A351K)*	this study
pBAD33-*capRel*^{SJ46}*(Y352A)*	arabinose inducible *capRel*^{SJ46}*(Y352A)*	this study
pBAD33-*capRel*^{SJ46}*(Y355A)*	arabinose inducible *capRel*^{SJ46}*(Y355A)*	this study
pBAD33-*capRel*^{SJ46}*(R78A)*	arabinose inducible *capRel*^{SJ46}*(R78A)*	this study
pBAD33-*capRel*^{SJ46}*(K311A)*	arabinose inducible *capRel*^{SJ46}*(K311A)*	this study
pBAD33-*capRel*^{SJ46}*(R314A)*	arabinose inducible *capRel*^{SJ46}*(R314A)*	this study
pBAD33-*capRel*^{SJ46}*(E319A)*	arabinose inducible *capRel*^{SJ46}*(E319A)*	this study
pBAD33-*capRel*^{SJ46}*(K346A)*	arabinose inducible *capRel*^{SJ46}*(K346A)*	this study
pBR322-*His*$_6$*-capRel*^{SJ46}	*capRel*^{SJ46} with N-terminal *His*$_6$*-tag*	this study
pBAD33-*gp57*	arabinose inducible *gp57*	this study
pBAD33-*gp57(L114P)*	arabinose inducible *gp57(L114P)*	this study
pBAD33-*gp57(I115F)*	arabinose inducible *gp57(I115F)*	this study
pBR322-*capRel*^{SJ46}*-FLAG*	*capRel*^{SJ46} with C-terminal *FLAG-tag*	this study
pBAD33-*gp57-HA*	arabinose inducible *gp57* with C-terminal *HA-tag*	this study
pBAD33-g*p57(L114P)-HA*	arabinose inducible *gp57(L114P)* with C-terminal *HA-tag*	this study
pBAD33-g*p57(I115F)-HA*	arabinose inducible *gp57(I115F)* with C-terminal *HA-tag*	this study
pBR322-*capRel*^{SJ46}*(L280Q)*	*capRel*^{SJ46}*(L280Q)* with native promoter	this study
pBR322-*capRel*^{SJ46}*(L280P)*	*capRel*^{SJ46}*(L280P)* with native promoter	this study
pBR322-*capRel*^{SJ46}*(L307A)*	*capRel*^{SJ46}*(L307A)* with native promoter	this study

pBR322-*capRel*^{SJ46}*(L280Q)-FLAG*	*capRel*^{SJ46}*(L280Q)* with C-terminal *FLAG-tag*	this study
pBR322-*capRel*^{SJ46}*(L280P)-FLAG*	*capRel*^{SJ46}*(L280P)* with C-terminal *FLAG-tag*	this study
pBR322-*capRel*^{SJ46}*(L307A)-FLAG*	*capRel*^{SJ46}*(L307A)* with C-terminal *FLAG-tag*	this study
pBAD33-*gp8*^{Bas4}	arabinose inducible *gp8*^{Bas4}	this study
pBAD33-*gp8*^{Bas5}	arabinose inducible *gp8*^{Bas5}	this study
pBAD33-*gp8*^{Bas8}	arabinose inducible *gp8*^{Bas8}	this study
pBAD33-*gp8*^{Bas4}*(Y113F)*	arabinose inducible *gp8*^{Bas4}*(Y113F)*	this study
pBAD33-*gp57(F113Y)*	arabinose inducible *gp57(F113Y)*	this study
pBAD33-*gp8*^{Bas8}*(F120L)*	arabinose inducible *gp8*^{Bas8}*(F120L)*	this study
pBAD33-*gp8*^{Bas8}*(I124F)*	arabinose inducible *gp8*^{Bas8}*(I124F)*	this study
pET24d-*gp57*	*gp57* expressed under the control of T7 promoter (VHp1172)	this study
pET24d-g*p57(L114P)*	*gp57(L114P)* expressed under the control of T7 promoter (VHp1369)	this study
pET24d-g*p57(I115F)*	*gp57(I115F)* expressed under the control of T7 promoter (VHp1370)	this study
pET24d-*His*$_{10}$*-SUMO*	*E. coli* codon-optimized *His*$_{10}$*-SUMO-tag* expressed under the control of T7 promoter (VHp167)	ref[59]
pET24d-*N-His*$_{10}$*-SUMO-capRel*^{SJ46}	*capRel*^{SJ46} with N-terminal *His*$_{10}$*-SUMO-tag* (VHp981)	this study
pMG25-*paSpo*	PaSpo SAH from *Salmonella* phage *SSU5* expressed under the control of IPTG inducible promoter $P_{A1/04/03}$ (VHp847)	ref[28]
DHFR Control Template	DHFR expressed under the control of T7 promoter	NEB
pET21a-*His*$_{10}$*-SUMO-gp57*	*gp57* with *His*$_{10}$*-SUMO-tag*	this study
pBR322-*capRel*^{Ebc}*-FLAG*	*capRel*^{Ebc} with C-terminal *FLAG-tag*	this study
pBR322-*chimera-FLAG*	*chimera* with C-terminal *FLAG-tag*	this study
pBR322-*capRel*^{EcHT}	*capRel*^{EcHT} with native promoter	this study

SI Table 4. Primers

Name	Purpose	Sequence (5'-3')
TZ-1	*capRel*[SJ46] into pBR322 backbone (with native promoter)	GCGTATCACGAGGCCCTTTCGTCTTCAAGA<br>ATTCTCATGTTAGTTACGTTAGGTAACATTA<br>TGGGTATGTTTCATTTTCATACCCATTGATT<br>TCTAGCTTGATTTGTTATTATATGTAACAAC<br>ACATTAATCATTTGAACTGGATGTTAGTAA<br>TGGGCAGTGAAGTATATCAAAGCCAAAAAT<br>GCGAGCTTAAATATTCGAAATCTCAAATAG<br>AAAAAGCAGCCAGAAAAATTAGACATGGT<br>TGTGAGGGCGCAGAGCGAGAAGAAGCAAT<br>TAAAATGATTCAAAATTTCCGCGAGCTTCA<br>TTTATATCCATTGATGCTAATGAAAAATCA<br>TCTTGATAGGGCTGCAAAAAAAGTTGATAA<br>AGAGAATAAGATTATTGTTGCAAGACGCCT<br>AAAAAGACTTAGCACAATAATTGATAAACT<br>GGAGCGTCCAAGTCTTGACGGCGGCGCAAC<br>AAATAATGCAATAGCCCTTACTAGAATGCA<br>GGATATTGGCGGATGTAGAGCTATAGTTAG<br>AAACATCGAACAACTAAAGAAACTAAAAG<br>ACCGACTAGTTAAAAGTCGCTCCAAGCATA<br>AAATCTTAAAGGAATATGACTATTTAACTC<br>CAAAGCCAAGCGGATATAGTGGCATTCACC<br>TTGCATATAGTTGTTTTGATGAAGAAAACG<br>GTAACAATCCGTGGAGTAAAACTAAAATCG<br>AAGTACAACTAAGAACAGAACTCCAACAT<br>GCTTGGGCAACAAGTCTGGAGATAATAGAT<br>ACACTTGAAAATATTAAGCTGAAAACTTCT<br>AATGAAGGTCACCCTGAGTGGAGAAGATTT<br>TTTTATCTTTCTGGATGCCTAGTTGCTCATG<br>ATGAAGGTGCCTGTATTCTTGATGATGAAA<br>CCATCAAAAATTATCAAACAGAGCTTAAAA<br>CATTGGAGGAGGCTTTATCTGTTCGCTCAA<br>AATTAAGTACATATACATTTGCAATGAAGC<br>TGACATCAGATGCAAACCTAAAGAAAAGC<br>CTGCCCAAAAACCATAATGGATTCTTTTTG<br>GTGAGGATGAGAAACGCCATAACAAAAAA<br>TGGCAAGGATACTGGAAAGTTTCTTGTTTC<br>TGTGAAACCTTTTAGGAAAAAAGAATCGGA<br>ACAAGCCCTCCAGGAACTTAATAAGGATGA<br>TGCGGATCCTGAAGTATTAATTGCGGTGCT<br>GCTTGCCACTAACAACATTAAATCTCTTAA<br>AAAGGCTTATCCTAACTATTTTGGGTCAAC<br>AAATCAGTTCGGGAGATTCCTTAGCAGACA<br>CATTGATACTGTGTAAATTCCTATGCCTGGC<br>TTAGCCAGGCATAGCCAAAGATTGGGCACC

		TCGCTAACGGATTCACCACTCCAAGAATTG GAGC
TZ-2	*capRel*^{Ebc} into pBR322 backbone (with native promoter) -fragment 1	CCTTTCGTCTTCAAGAATTCTCATGTTTACC CTACATACATCATTATTGACGGTATCAAGA TCAGCCGCTCCCGCTTGTCATTTGCCTTTCA GTTGGAGTTAGCAAAAGCCCTTAAAATTTA TGAAGAAAATCATACGTAAGTAGCATAAAT GACTTGAGTTTTCAGCCAGTATGCATTCTCA TGCGTACATACATGGCAATAATATTAACGG GAGGATATCGGGAATGGGCAATGAAGTTTA TGAAAGCCAGAAGTGTACGCTTAAATTTAG TAAGACCCTTATTGATAAGTCCGCGCAGTC GATCCGTCATGGGTGTGATGGTGAGCAAAA AGCACAGGCTATTGCTATGATCCAGAACTT CCGTGAACTTCACCTTTATCCGTTGATGTTA ATGAAAAATCACTTAGCCCGTGCCGCGGCA AAGATTGACAAAAAGACTATTGTCGCACGC CGCCTTAAGCGTTTAAGTACCATCATTGAT AAATTGGAACGCCCCACCTTAGACAATGGA AAAACCAGCAATGCAATCCGTGTAACACGC ATGCAGGATATTGGGGGTTGTCGTGCAATC GTTAAAAACATCGAACAGCTTAAAGAATTG CGCGAGCGCCTGGTTAAATCCCGCAGCGTA CATAAAATCATCAAAGAGAGTGATTATTTG AC
TZ-3	*capRel*^{Ebc} into pBR322 backbone (with native promoter) -fragment 2	AGAGAGTGATTATTTGACTCCAAAAGAGTC GGGGTACGGTGGTATCCACTTAGTATATAG CTGTTTTGATTCCAGTGATGACAATTACCCA TGGAAAAAGACGAAGATCGAGGTACAACT TCGTACGCAGTTGCAGCACGCATGGGCAAC ATCTTTAGAGATCATCGATACGTTAGAGAA CATTAAGTTGAAAACCTCTAGCGAGGGGCA CGAAGATTGGCGCCGCTTTTTTTATTTGAGC GGGTGTCTGGTAGCTCACGATGAGAAGGCC TGCGTGCTGGACAAGGAGGTAGTAAAGGA ATACCAAAAGGAGTTGAAAGCCTTAGCGG AGCATCTGTCTGTCATTAGCAAGTTGGGTA GTTATGTGTTTTCACTTAGCGTGACTTCACA AAACGAACTTAAAAACAAAATTCCAAAAA ACCATAAGGGACATTATCTGGTCACTATGA AGCGCGTGAAGGACGAACAGCAGCCTGGG AAAATTAAGTTTGAGGTGTCCGTGACTCCG TACCGTATTAACCAAGCCGATGAGGCCTTA GAGGCATTGAAACGTGATGATGCAAATCCG GAAGTGCCCATCGCTGTGTTGCTTGCGACC GAATCCGTGAAATCACTTAAAAAGGCATAC CCAAATTACCTGGGCTCCACTAACCAGTTT

		GCACGCTTTTTAAGCAAGCATATTAAGTAA GGCACCTCGCTAACGGATTCACCAC
TZ-4	*capRel*^{Kp} into pBR322 backbone (with native promoter) -fragment 1	CCTTTCGTCTTCAAGAATTCTCATGTCATCC TCTGTTACACGGCGAGTTCCACCATGGGCA CAGCTTACGTTTGATTTAATGATTTATTCCA GCCCCTTCATGCGAGGGGGTTGGGCTAAGT CAGGCAAAACGAGACATGTTGCACATTGTA GTGTGATTGCATTGTATCAAAAATATCAGG AGGTTATAGTGCCAGAACTCAAGCTATTAG ATGAAGGCAGTCACATGGCTAATGCACAAT ACGAGAAGGGGAAGATTGTTTTAGCTTACT CGAAAAATCAAGTTAAGAAAGCGGGCGAA TGTATTCGTAAAGAGAACGGAGATATTGAA AAGTCAATTGAAATCATTCAGCAATATCGC GCAGCACATTTATATCCGTTAATGATCGTG AAAAATTTAGTTTGGAAACATGCACAGAAG ATCAACAAAAACGCGATCATTGCACGTCGC TTAAAACGTCTTCCTACAATCATTGATAAA TTGCGTCGCAAAACCCTGGACGGGGTGACG GCTAATTCAATCGCAGTCACTCGCATGTCG GATATCGGTGGATGTCGTGTAATCGTGGAA GACCGTTTGCAACTGCTGCTTCTGGATTCCT CCTTAGATAAAAGTCGTACCACGCATAAGT CAAAGGTGAAGGATTACATTAAGTCGCCGA AACCTACTGGGTACCGTG
TZ-5	*capRel*^{Kp} into pBR322 backbone (with native promoter) -fragment 2	GCCGAAACCTACTGGGTACCGTGGGATTCA TCGCATCTATTCCTGCTATGACCGCGACGA AGCGCACAAATGGAAAGGGTTCGACATCG AGGTGCAGTTACGTACAAAGTTGCAACATC TTTGGGCAACTACAGTAGAAGTAGTAGACT TGTGCGAGGGCCGCAGCTTAAAGACCAACC CGTTCGAATCGAATCCTTCGTGGATTGAGT TCTTCCGCCTTATGTCAGAATTTATCGCCGA TGAGGAAGGCTTTATTTACCTTTCCCCCCAG GACAAGAATTTACTGAAGACTCGCTTGATC TCTTTGAATAATAAGCTGAATGCGATTGAC AAACTGTTGTCTTTTAACCGTCTTTTTTCTG ATAAAAAGATTAACCTGTCTCAACGTAAGT CCGGGTATGTAATCATTGCCATTAAAGGTA ATTCTATCTATTATAAGTTTTTCAGCCCAAC TCAAAAACACAAAGCCGTAGAACAATACTC GATTATTGAAAAGGACGATGACTATAATGG CTTGTTCGTCGAGATGGACGATATTCGCAA ACTGTCATATGCGTATCCCAACTACTTAATT GATACGCGTTTTTTCATTGACAAGTTTGAAT TATACACCAATACAAATTATTGGGTCAAGC

		CACGTTAAGGCACCTCGCTAACGGATTCACCAC
TZ-6	linearize pBR322	GGCACCTCGCTAACGGAT
TZ-7	linearize pBR322	ACATGAGAATTCTTGAAGACGAAAGG
TZ-8	generate Y155A in *capRel*^{SJ46}	GCAAGTGGCATTCACCTTGCATATAGTTG
TZ-9	generate Y155A in *capRel*^{SJ46}	TCCGCTTGGCTTTGGAGTTAA
TZ-10	generate Y153A in *capRel*^{Ebc}	GTCGGGGGCGGGTGGTATCCACTTAGTATATAGCT
TZ-11	generate Y153A in *capRel*^{Ebc}	CCACCCGCCCCCGACTCTTTTGGAGTCAA
TZ-12	linearize pBAD33	AAGCTTGGCTGTTTTGGC
TZ-13	linearize pBAD33	CTCGAATTCGCTAGCCCAAA
TZ-14	*capRel*^{SJ46}*(1-272)* and *capRel*^{SJ46} into pBAD33	TACCCGTTTTTTTGGGCTAGCGAATTCGAGTGAACTGGATGTTAGTAATGGGCAGT
TZ-15	*capRel*^{SJ46}*(1-272)* into pBAD33	CTTCTCTCATCCGCCAAAACAGCCAAGCTTTTATGATGTCAGCTTCATTGCAAATGT
TZ-16	linearize pEXT20	CGGGGATCCTCTAGAGTCGAC
TZ-17	linearize pEXT20	GGTACCGAGCTCGAATTCTGTTTC
TZ-18	*capRel*^{SJ46}*(273-373)* into pEXT20	AATTCGAGCTCGGTACCGGGAGGATATCGGGAATGGATGCAAACCTAAAGAAAAGCCTGC
TZ-19	*capRel*^{SJ46}*(273-373)* into pEXT20	GCAGGTCGACTCTAGAGGATCCCCGTTACACAGTATCAATGTGTCTGCTAAGGA
TZ-20	linearize pBR322-*capRel*^{SJ46} to generate pBR322-*capRel-chimera*	GTGTTGCTTGCGACTAACAACATTAAATCTCTTAAAAAGGCT
TZ-21	linearize pBR322-*capRel*^{SJ46} to generate pBR322-*capRel-chimera*	TAAGTTCGTTTTGTGATGTCAGCTTCATTGCAAATGTATATGTACTTAATT
TZ-22	insert *capRel*^{Ebc}(270-339) to generate pBR322-*capRel-chimera*	AAGCTGACATCACAAAACGAACTTAAAAACAAAATTCCAAAAAACC
TZ-23	insert *capRel*^{Ebc}(270-339) to generate pBR322-*capRel-chimera*	GTGTTGCTTGCGACTAACAACATTAAATCTCTTAAAAAGGCT
TZ-24	*capRel*^{SJ46} into pBAD33	CTTCTCTCATCCGCCAAAACAGCCAAGCTTTTACACAGTATCAATGTGTCTGCTAAG
TZ-25	generate A77K in *capRel*^{SJ46}	TATTGTTAAAAGACGCCTAAAAAGACTTAG
TZ-26	generate A77K in *capRel*^{SJ46}	CGTCTTTTAACAATAATCTTATTCTCTTTATCA
TZ-27	generate R116A in *capRel*^{SJ46}	CGGATGTGCGGCTATAGTTAGAAACATCGAACAAC
TZ-28	generate R116A in *capRel*^{SJ46}	ATAGCCGCACATCCGCCAATATCCTGC
TZ-29	generate V338A in *capRel*^{SJ46}	AATTGCGGCGCTGCTTGCCACTAACAACA
TZ-30	generate V338A in *capRel*^{SJ46}	AGCAGCGCCGCAATTAATACTTCAGGATCCGC

TZ-31	generate L339A in *capRel*^{SJ46}	TGCGGTGGCGCTTGCCACTAACAACATTAAATCTC
TZ-32	generate L339A in *capRel*^{SJ46}	GCAAGCGCCACCGCAATTAATACTTCAGGATCC
TZ-33	generate A341K in *capRel*^{SJ46}	GCTGCTTAAAACTAACAACATTAAATCTCTTAAAA
TZ-34	generate A341K in *capRel*^{SJ46}	TTAGTTTTAAGCAGCACCGCAATTAA
TZ-35	generate A351K in *capRel*^{SJ46}	TAAAAAGAAATATCCTAACTATTTTGGGTCAAC
TZ-36	generate A351K in *capRel*^{SJ46}	GGATATTTCTTTTTAAGAGATTTAATGTTGTT
TZ-37	generate Y352A in *capRel*^{SJ46}	AAAGGCTGCGCCTAACTATTTTGGGTCAACAAATC
TZ-38	generate Y352A in *capRel*^{SJ46}	TTAGGCGCAGCCTTTTTAAGAGATTTAATGTTG
TZ-39	generate Y355A in *capRel*^{SJ46}	TCCTAACGCGTTTGGGTCAACAAATCAGTTCGGG
TZ-40	generate Y355A in *capRel*^{SJ46}	CCAAACGCGTTAGGATAAGCCTTTTTAAGAG
TZ-41	construct pBR322-*His*$_6$-*capRel*^{SJ46}	TCACCATCACCACGGCAGCAGCGGCATGGGCAGTGAAGTATATCAAAG
TZ-42	construct pBR322-*His*$_6$-*capRel*^{SJ46}	CCGTGGTGATGGTGATGATGCATTACTAACATCCAGTTCAAATGATT
TZ-43	*gp57* into pBAD33	TACCCGTTTTTTTGGGCTAGCGAATTCGAGATGGCTAAAAAATATGATGAACTAGATGC
TZ-44	*gp57* into pBAD33	TTCTCTCATCCGCCAAAACAGCCAAGCTTTTAAACTGACTTTTTCAAGCCAGTAATAAGC
TZ-45	construct pBR322-*capRel*^{SJ46}-*FLAG*	GCGATTACAAGGATGACGATGACAAATAAATTCCTATGCCTGGCTTAGC
TZ-46	construct pBR322-*capRel*^{SJ46}-*FLAG*	CATCCTTGTAATCGCCGCTGCTGCCCACAGTATCAATGTGTCTGCTAAGG
TZ-47	construct pBAD33-*gp57-HA*	CTACCCGTATGATGTGCCGGACTATGCATAAAAGCTTGGCTGTTTTGGCGG
TZ-48	construct pBAD33-*gp57-HA*	ACATCATACGGGTAGCCGCTGCTGCCAACTGACTTTTTCAAGCC
TZ-49	generate L280Q in *capRel*^{SJ46}	GAAAAGCCAGCCCAAAAACCATAATGGATTC
TZ-50	generate L280Q in *capRel*^{SJ46}	TTGGGCTGGCTTTTCTTTAGGTTTGCATCTG
TZ-51	generate L280P in *capRel*^{SJ46}	GAAAAGCCCGCCCAAAAACCATAATGGATTC
TZ-52	generate L280P in *capRel*^{SJ46}	TTGGGCGGGCTTTTCTTTAGGTTTGCATCTG
TZ-53	generate L307A in *capRel*^{SJ46}	AAAGTTTGCGGTTTCTGTGAAACCTTTTAGGAAAA
TZ-54	generate L307A in *capRel*^{SJ46}	GAAACCGCAAACTTTCCAGTATCCTTGCCA

TZ-55	$gp8^{Bas4}$ into pBAD33	TACCCGTTTTTTTGGGCTAGCGAATTCGAG ATGGCTAAAAAATATGATGAACTAGATGCT
TZ-56	$gp8^{Bas4}$ into pBAD33	CTTCTCTCATCCGCCAAAACAGCCAAGCTT TTACTCAGCTTTTTTCAAGCCG
TZ-57	$gp8^{Bas5}$ into pBAD33	TACCCGTTTTTTTGGGCTAGCGAATTCGAG ATGACAAAGAAAAAATATGATGAGCTAGA C
TZ-58	$gp8^{Bas5}$ into pBAD33	CTTCTCTCATCCGCCAAAACAGCCAAGCTT TTACGCAGCCTTTTTCAAGCCA
TZ-59	$gp8^{Bas8}$ into pBAD33	CCCGTTTTTTTGGGCTAGCGAATTCGAGAT GAGGGAAAATATTATGTCTAAAGAAATGA A
TZ-60	$gp8^{Bas8}$ into pBAD33	CTTCTCTCATCCGCCAAAACAGCCAAGCTT TCAGCCTACTACAAGACCTTTAATCA
TZ-61	generate Y113F in $gp8^{Bas4}$	TAACGCTTTTCTGATCTCCATTGATGAGATT AAAG
TZ-62	generate Y113F in $gp8^{Bas4}$	ATCAGAAAAGCGTTACCCAGGCGGAA
TZ-63	generate F113Y in *gp57*	TAACGCTTATCTGATCTCCATTGATGAGATT AAAG
TZ-64	generate F113Y in *gp57*	ATCAGATAAGCGTTACCCAGGCGGAA
TZ-65	*gp57* cloned into pET24d	CTTTAAGAAGGAGATATACCATGGCTAAAA AATATGATGAAC
TZ-66	*gp57* cloned into pET24d	GCCGGATCTCAGTGGTGGTGTTAAACTGAC TTTTTCAAGC
TZ-67	*gp57*	CTGTGTGAAATTGTTATCCGAAAAATAAGG AGGAAAAAAAAATGGCTAAAAAATATGAT GAACTAGATGCTACGATTGTAGCGAATCAT TTGCAGATTCAGGGTGTCAAGACCGACGCT TCTGATATGGGTATTTGGACCGCTCAAGAG CTACACAAGATCCGCTCAACCGCATACGAG AAAGAATATCCGGCAGGTTCCGCGCTTCGC GTATTCCCTGTAACAAACGAGCTTTCTGAT ACTGATAAGACTTTTGAGTATCAGACTTTT GATAAGGTTGGCTACGCGAAAATTATCGCC GACTACACCGACGATCTGCCGACCGTGGAC GCGCTGATGACTTCTGAATTTGGCAAGGTG TTCCGCCTGGGTAACGCTTTTCTGATCTCCA TTGATGAGATTAAAGCAGGTCAGCGAACTG GCAAGAGCCTGTCAACTCGCAAGGCTAACG CCGCGCAAAATGCACATGATCAGCTGATTA ACTTCCTGGTGTTCAAAGGTTCCAAGCCTC ATAAGATCGTTTCCGTTTTCGATCATCCTAA CCTTACGAAAATTGTTTCTAAAGGATGGAT GAGCCAGGATGGAAACACCAAGTTCCCTGA TGTGGCAAGCGATGAACTGGAGGCTGCAAT CGAAACGATCGAGGAAGTAACCAAAGGTC AGCACCGAGCGACTAACATCCTGATCCCGC

		CGTCCATGCGCAAAGTCCTGACGGTTCGAATGGAAAATACCACTGAAAGTTATCTGGAATACTTCCAGAAGCAAAACGGCGGCATCACTATCGACTCTATCGCAGAGCTTGAGGATATTGACGGCAAAGGTACTAAAGGTTGCTTGGTTTACGAAAAAGATCCAATGAACATGAGCATTGAGATTCCAGAAGCGTTTAACATGCTTCCGGCGCAGCCAAAAGACCTTCATTTCAAGGTTCCTTGCACTTCCAAGTGTACTGGCCTTACGATTTACCGTCCGTTTACGATGGTGCTTATTACTGGCTTGAAAAAGTCAGTTTAAGCTTGGACTCCTGTTGATAG
TZ-68	Linearized pET24d plasmid without His_6-tag	CACCACCACTGAGATCCGGC
TZ-69	Linearized pET24d plasmid without His_6-tag	GGTATATCTCCTTCTTAAAGTTAAACAAAATTATTTC
TZ-70	*gp57(L114P)* cloned into pET24d	ATGGAGATCGGAAAAGCGTTACCCAGGCGGAAC
TZ-71	*gp57* mutants cloned into pET24d	CAGCAACGCGGCCTTTTTACG
TZ-72	*gp57(L114P)* cloned into pET24d	AACGCTTTTCCGATCTCCATTGATGAGATTAAAGCAGGTCAG
TZ-73	*gp57* mutants cloned into pET24d	GTAAAAAGGCCGCGTTGCTGG
TZ-74	*gp57(I115F)* cloned into pET24d	CTCATCAATGGAAAACAGAAAAGCGTTACCCAGGCGG
TZ-75	*gp57(I115F)* cloned into pET24d	TTCTGTTTTCCATTGATGAGATTAAAGCAGGTCAGC
TZ-76	$capRel^{SJ46}$ cloned into pET24d-*N-*His_{10}*-SUMO*	ATCGCGAACAGATTGGTGGTGGCAGTGAAGTATATCAAAGCCAAAAATGCGAGC
TZ-77	$capRel^{SJ46}$ cloned into pET24d-*N-*His_{10}*-SUMO*	GGTGGTGGTGGTGCTCGAGTTCTCATCCGCCAAAACAGCC
TZ-78	Linearized pET24d-*N-*His_{10}*-SUMO*	ACCACCAATCTGTTCGCGATGAGC
TZ-79	Linearized pET24d-*N-*His_{10}*-SUMO*	ACTCGAGCACCACCACCAC
TZ-80	generate R78A in $capRel^{SJ46}$	TGTTGCAGCGCGCCTAAAAAGACTTAGCACA
TZ-81	generate R78A in $capRel^{SJ46}$	AGGCGCGCTGCAACAATAATCTTATTCTCTTT
TZ-82	generate K311A in $capRel^{SJ46}$	TTCTGTGGCGCCTTTTAGGAAAAAAGAATCGGAAC
TZ-83	generate K311A in $capRel^{SJ46}$	AAAGGCGCCACAGAAACAAGAAACTTTCCAG
TZ-84	generate R314A in $capRel^{SJ46}$	ACCTTTTGCGAAAAAAGAATCGGAACAAGCCCTC

TZ-85	generate R314A in *capRel*^{SJ46}	TTTTTCGCAAAAGGTTTCACAGAAACAAGAAAC
TZ-86	generate E319A in *capRel*^{SJ46}	AGAATCGGCGCAAGCCCTCCAGGAACTTAATAAG
TZ-87	generate E319A in *capRel*^{SJ46}	GCTTGCGCCGATTCTTTTTCCTAAAAGGTTTC
TZ-88	generate K346A in *capRel*^{SJ46}	CAACATTGCGTCTCTTAAAAAGGCTTATCCTAAC
TZ-89	generate K346A in *capRel*^{SJ46}	AGAGACGCAATGTTGTTAGTGGCAAGCAG
TZ-90	construct pBR322-*capRel*^{Ebc}-*FLAG*	GCGATTACAAGGATGACGATGACAAATAAGGCACCTCGCTAACGGAT
TZ-91	construct pBR322-*capRel*^{Ebc}-*FLAG*	CATCCTTGTAATCGCCGCTGCTGCCCTTAATATGCTTGCTTAAAAAGCGTGC
TZ-92	*capRel*^{EcHT} into pBR322 backbone (with native promoter) fragment 1	CCTTTCGTCTTCAAGAATTCTCATGTCCGCTTACCCCACATACATCATTATTGACGGTATCAAGATCAGTCGATCCCGCCTGTCATTTGCCTTTCAGTTGGAGTTAGCGAAAGCCCTTAAAATTTATGAAGAAAATCAGAAGTAGGTTACATAAATGACTTGAATTTTCAGCCCATTTGCATTCTCATATTTGCTTACACGGCAATAAGTTAACGGAGCATTGGAAATGGGTAATGAAGTGTATGAATCACAGAAGTGTACGCTTAAATATTCGAAATCCCAGATCGATAAGGCGGCCCAGCTTGTACGCCACGGTTGTAATGGAGCGGAGCGCGAGGAAGCGATTGCCATGATCCAGAACTTCCGTGAGTTGCACCTGTATCCTCTGATGCTTATTAAAAATCATCTGGCGCGTGCTAGCGTACGTGTCGACAAAAAGATCATCGTAGCGCGTCGTTTGAAGCGTTTAAGCACTATCATTGATAAGTTGGAACGTTCCTCGTTAGACGGCGGTAAGACCACGAATTCCATCAAAATGACCCGCATGCAGGACATTGGCGGATGCCGTGCTATTGTTAAGAATATCGAACAGTTAAAGCTGTTACGCGACCGTCTTATCAAGTCACGCTCCGTTCACAAAGTCATCCGTGAATCTAATTACTTAACACCAAAAGAGTCCGGGTATGGAGGTATCCATTTAATTTACTCTTGTTTCGACGGGTCGG
TZ-93	*capRel*^{EcHT} into pBR322 backbone (with native promoter) fragment 2	TAATTTACTCTTGTTTCGACGGGTCGGAAGATAAGTTTCCGTGGAAAAAGACAAAAATTGAGGTACAATTGCGTACTGAGCTGCAACATGCATGGGCGACCTCATTGGAGATTATCGATACACTTGAAAACATCAAGTTGAAGACTAGTAATGAGGGCCATGCAGAATGGCGCCGTTTTTTCTACATCGCTGGATGTCTTGTTGCACACGACGAGCGTGCTTGTATCTTATCTAAGGATAAGGTGGAAGAATACCAAAAAGAGTTAAGCACCTTGGAAAGTGAACTTGACGTCCGCAAG

		AAGTTAGCTTCCTACGTGTTCTCGCTTTCTG TGACATCAGATGATAAGTTAATCAAAAAGC TTCCGAAGAATTACCGTGGTCATTTCTTGGT AACAATGAAACGTGAGCCGGTTGATCCAGA CAATAAGGACAAGATTCGCTTCGCGGTTGC ACTGGAAGCCTTCCGTATTAAAGAAGCGGA CGAGGCATTAGAGGCGTTGAAACGTGACG ACGCTGACCCCGAAGTGCTGATTGCTGTCT TGTTAGCGACCGACAATATCAAGTCGCTTA AAAAGGCGTATCCCAACTATTTGGGGTCTA CGTCCCAGTTTGACAAGTTCTTAAACAAGC ACATTAAATAAGGCACCTCGCTAACGGATT CACCAC
TZ-94	error-prone PCR insert of *cap-Rel*^{SJ46} C-terminus	GGCAAGCAGCACCGCAAT
TZ-95	error-prone PCR insert of *cap-Rel*^{SJ46} C-terminus	ACATATACATTTGCAATGAAGCTGACATCA
TZ-96	linearize pBR322-*capRel*^{SJ46} for error-prone PCR	ATTGCGGTGCTGCTTGCC
TZ-97	linearize pBR322-*capRel*^{SJ46} for error-prone PCR	TGATGTCAGCTTCATTGCAAATGTATATGT
