# Supplementary material for: Direct activation of a bacterial innate immune system by a viral capsid protein
Source: Nature. 2022 Nov 16;612(7938):132–40. doi: 10.1038/s41586-022-05444-z (PMC9712102; doi:10.1038/s41586-022-05444-z)
Supplement: Supplementary file 2 — Reporting Summary [file 41586_2022_5444_MOESM2_ESM.pdf]

nature portfolio

Corresponding author(s): Hauryliuk, Garcia-Pino, Laub

Last updated by author(s): Sep 28, 2022

Reporting Summary

Nature Portfolio wishes to improve the reproducibility of the work that we publish. This form provides structure for consistency and transparency in reporting. For further information on Nature Portfolio policies, see our Editorial Policies and the Editorial Policy Checklist.

Statistics

For all statistical analyses, confirm that the following items are present in the figure legend, table legend, main text, or Methods section.

n/a	Confirmed	
☐	☒	The exact sample size (*n*) for each experimental group/condition, given as a discrete number and unit of measurement
☐	☒	A statement on whether measurements were taken from distinct samples or whether the same sample was measured repeatedly
☐	☒	The statistical test(s) used AND whether they are one- or two-sided<br>*Only common tests should be described solely by name; describe more complex techniques in the Methods section.*
☒	☐	A description of all covariates tested
☒	☐	A description of any assumptions or corrections, such as tests of normality and adjustment for multiple comparisons
☐	☒	A full description of the statistical parameters including central tendency (e.g. means) or other basic estimates (e.g. regression coefficient) AND variation (e.g. standard deviation) or associated estimates of uncertainty (e.g. confidence intervals)
☐	☒	For null hypothesis testing, the test statistic (e.g. *F*, *t*, *r*) with confidence intervals, effect sizes, degrees of freedom and *P* value noted<br>*Give P values as exact values whenever suitable.*
☒	☐	For Bayesian analysis, information on the choice of priors and Markov chain Monte Carlo settings
☒	☐	For hierarchical and complex designs, identification of the appropriate level for tests and full reporting of outcomes
☒	☐	Estimates of effect sizes (e.g. Cohen's *d*, Pearson's *r*), indicating how they were calculated

Our web collection on statistics for biologists contains articles on many of the points above.

Software and code

Policy information about availability of computer code

Data collection	Biotek Gen5 v. 3.02 for growth curve data.<br>NCBI BLAST for homology searches.<br>PHASTER web server for searching prophage regions.<br>Consurf 2016 web server for identifying additional homologs.<br>MAFFT L-INS-i v7.453 for aligning sequences.<br>FlaGs v1.2.6 for gene neighborhood analysis.
Data analysis	Phenix 1.20.1-4487, Coot 0.9.8.1, BUSTER / TNT 2.10.4, Fiji 2.1.0/1.53c, PyMOL 2.4.2, Prism 9.4.0, Geneious 2022.0.2, MMSeqs v13.45111

For manuscripts utilizing custom algorithms or software that are central to the research but not yet described in published literature, software must be made available to editors and reviewers. We strongly encourage code deposition in a community repository (e.g. GitHub). See the Nature Portfolio guidelines for submitting code & software for further information.

Data

Policy information about availability of data

All manuscripts must include a data availability statement. This statement should provide the following information, where applicable:

- Accession codes, unique identifiers, or web links for publicly available datasets
- A description of any restrictions on data availability
- For clinical datasets or third party data, please ensure that the statement adheres to our policy

Structural data in this study are available in PDB (7ZTB). HDX data raw data can be accessed at: doi.org/10.6084/m9.figshare.19745089. Sequencing data are available in the Sequence Read Archive (SRA) under BioProject PRJNA837951. All other data are available in the manuscript or the supplementary materials. Source

data for graphs in Fig. 1-5 and Extended Data Fig. 4 are available. Other previously published structures are available in PDB (5DEC, 6S2T, 2LVH). UniRef90 database is publicly available. Reference phage genomes are publicly available: SECΦ27(NC_047938.1), Bas04 (MZ501069.1), Bas08(MZ501059.1). Materials including strains and plasmids are available upon reasonable request.

Field-specific reporting

Please select the one below that is the best fit for your research. If you are not sure, read the appropriate sections before making your selection.

☒ Life sciences ☐ Behavioural & social sciences ☐ Ecological, evolutionary & environmental sciences

For a reference copy of the document with all sections, see nature.com/documents/nr-reporting-summary-flat.pdf

Life sciences study design

All studies must disclose on these points even when the disclosure is negative.

Sample size	Sample sizes were chosen based on the number needed to reliably determine differences between groups. Given large effect sizes, we chose to replicate experiments in triplicate as is routine to simply indicate reproducibility.
Data exclusions	No data were excluded from analysis.
Replication	All experimental findings were repeated at least twice. All reported results were successfully reproduced.
Randomization	No experimental groups or control groups were subjectively chosen and there are no covariates to control for as experiments were done in isogenic strains. No randomization is required.
Blinding	Blinding was not required because all data were obtained objectively and had strong effect sizes over multiple independent replicates and raw data are reported in the manuscript.

Reporting for specific materials, systems and methods

We require information from authors about some types of materials, experimental systems and methods used in many studies. Here, indicate whether each material, system or method listed is relevant to your study. If you are not sure if a list item applies to your research, read the appropriate section before selecting a response.

Materials & experimental systems

n/a	Involved in the study	
☐	☒	Antibodies
☒	☐	Eukaryotic cell lines
☒	☐	Palaeontology and archaeology
☒	☐	Animals and other organisms
☒	☐	Human research participants
☒	☐	Clinical data
☒	☐	Dual use research of concern

Methods

n/a	Involved in the study	
☒	☐	ChIP-seq
☒	☐	Flow cytometry
☒	☐	MRI-based neuroimaging

Antibodies

Antibodies used	6x-His Tag Monoclonal Antibody (HIS.H8) (Invitrogen, catalog # MA1-21315), HA-tag (C29F4) rabbit mAb (Cell Signaling Technology #3724), anti-GyrA polyclonal (Inspiralis, PA001), (FLAG) DYKDDDDK Tag (D6W5B) Rabbit mAb (Cell Signaling Technology, #14793)
Validation	Antibodies have been validated based on manufacturer's website:<br>anti-His: validated by "western blot analysis of His-tagged protein in E. coli lysates".<br>anti-HA: validated by "western blot analysis of extracts from HeLa cells, untransfected or transfected with either HA-FoxO4 or HA-Akt3".<br>anti-GyrA polyclonal: reference Ey, P.L., S.J., & Jenkin, C.R. (1978).<br>anti-FLAG: validated by "western blot analysis of extracts from 293T cells, mock transfected (-) or transfected with DYKDDDDK-GFP".<br>In addition, all western blotting experiments include controls with untagged proteins as internal validation for antibodies.
